# Epinephrine minimizes the use of bipolar coagulation and preserves ovarian reserve in laparoscopic ovarian cystectomy: a randomized controlled trial

**DOI:** 10.1038/s41598-020-77781-w

**Published:** 2020-12-01

**Authors:** Eun Young Park, Kyu-Hee Hwang, Ji-Hee Kim, San-Hui Lee, Kyu-Sang Park, Seong Jin Choi, Seung-Kuy Cha

**Affiliations:** 1 Department of Obstetrics and Gynecology, Yonsei University Wonju College of Medicine, 20 Ilsan-ro, Wonju, Gangwondo 26426 Republic of Korea; 2 Department of Physiology, Department of Global Medical ScienceMitohormesis Research Center, Institute of Mitochondrial Medicine, Yonsei University Wonju College of Medicine, 20 Ilsan-ro, Wonju, Gangwondo 26426 Republic of Korea

**Keywords:** Infertility, Outcomes research, Randomized controlled trials

## Abstract

We propose a novel method, the epinephrine compression method (Epi-pledget), as a hemostasis method for ovarian cystectomy. A total of 179 patients undergoing laparoscopic ovarian cystectomy with stripping were randomly allocated into three groups: the bipolar coagulation group, the Epi-pledget group, and the coagulation after Epi-pledget (Epi & Coagulation) group. Serum anti-Müllerian hormone (AMH) levels and antral follicle count (AFC) by ultrasonography were measured to determine the preservation of ovarian function. To evaluate the postoperative ovarian cellular proliferative activity and tissue damage in a mouse model, we operated on the ovaries of mice with an artificial incision injury and applied two hemostatic methods: coagulation and Epi-pledget. Eight weeks after surgery, the AMH rate significantly decreased in the bipolar coagulation group compared with the Epi-pledget group. The AFC decline rate was also significantly greater in the coagulation group than the Epi-pledget group. Specifically, patients with endometrioma had a significantly greater decline of serum AMH in the coagulation group than the Epi-pledget group. In a histopathological analysis in mice, the Epi-pledget group showed ameliorated fibrotic changes and necrotic findings in the injured lesion compared with the bipolar coagulation group. The Epi-pledget method for ovarian stripping has an additional benefit of maximizing the preservation of the ovarian reserve, especially for the endometriotic ovarian cyst type.

## Introduction

Surgical laparoscopy is the most common technique for the treatment of benign ovarian cysts. Laparoscopic ovarian cystectomy has been demonstrated to improve post-operative fecundability and reduce the recurrence rate compared with fenestration and coagulation of the cyst wall^1^. However, laparoscopic cystectomy is associated with a risk of surgical injury to the remnant normal ovarian tissue^1^. The radical laparoscopic surgical approach resulted in an improvement of several disease-related symptoms and quality of life indices^2^. Balancing the surgical radicality between the success of treatment (reduction of pain and recurrence) and the ovarian reserve is a challenging issue. Another crucial issue in laparoscopic ovarian cystectomy is how to reduce the damage and bleeding to preserve ovarian function. Many factors have been reported to affect the ovarian reserve, such as different surgical techniques for the excision (traditional stripping and combined excisional/ablative techniques)^3^, mode of hemostasis (suture, compression, and cauterization)^4^, operational method (cystectomy vs. wedge resection), and cyst characteristics (size, location, and pathologic types)^5,6^. Notably, hemostasis by bipolar coagulation, which is commonly used, can result in thermal damage to healthy ovarian follicles and reduce the ovarian reserve^7–9^.

Pharmacological intervention is an alternative hemostasis method for minimizing tissue damage. Vasopressin is commonly used for hemostasis that occurs from the stripping of ovarian cysts and myomectomy^10^. However, intra-operative local injections of vasopressin frequently cause cardiopulmonary complications, such as cardiac arrest, hypotension, and pulmonary edema^11–13^. Therefore, we propose an alternative pharmacological intervention for hemostasis, epinephrine pledget compression (Epi-pledget). The Epi-pledget is widely used in endoscopic sinonasal surgery for local vasoconstriction and bleeding control^14^, subsequently reducing blood leakage to the maxillary sinus and subcutaneous tissue after canine fossa puncture^15^. A recent study showed that topical epinephrine applied in prostate surgery facilitated post-operative hemostasis^16^. Intravenous low-dose epinephrine was used in total hip arthroplasty, resulting in a reduction of both bleeding and inflammatory reaction^17^. In addition, an epinephrine injection successfully produced hemostasis for bleeding of colonic diverticula in colonoscopy^18^. The present study evaluated the efficacy of the Epi-pledget on hemostasis to minimize subsequent complications including decreased ovarian reserve, tissue damage and bleeding in laparoscopic cystectomy.

## Results

Table 1 shows the baseline characteristics of all participants in the three groups. The mean age, BMI, a maximum diameter of ovarian cysts, and hemoglobin level did not differ among the three groups. Also, there was no difference in preoperative anti-Müllerian hormone (AMH) and antral follicle count (AFC) levels among the three groups (Table 1, Supplementary Table S1).

**Table 1:** Preoperative patient baseline characteristics.

	Bipolar Cauterization group (n = 65)	Epi-pledget group (n = 62)	Epi-pledget and Cauterization group (n = 52)	*P* value
Age (years)	32.6 ± 9.1	32.8 ± 8.0	32 ± 9.6	0.911
Body mass index (kg/m^2^)	21.5 ± 3.7	21.9 ± 5.1	23.6 ± 6.2	0.074
**Parity (n (%))**				
Nulliparous	49 (75%)	51 (82%)	34 (65%)	
Parous	16 (15%)	11 (18%)	18 (35%)	
Maximum diameter of ovarian cyst (cm)	6.3 ± 2.3	6.9 ± 1.7	6.8 ± 2.2	0.304
Preoperative Hb level (mg/dL)	12.13 ± 1.1	12.31 ± 1.2	12.62 ± 2.2	0.427
Preoperative AMH level (ng/dL)	5.16 (2.54–7.26)	5.47 (2.6–7.66)	3.90 (2.89–5.9)	0.057
Preoperative AFC (n)	2.52 ± 1.08	2.21 ± 0.94	2.25 ± 1.04	0.211
**Type of ovarian cyst (n)**				
Endometriotic cyst	20	26	13	
Mature cystic teratoma	3	2	3	
Serous cystadenoma	11	11	2	
Mucinous cystadenoma	31	23	34	

Data are presented as mean ± standard deviation, median (interquartile ranges), or frequencies (percentages).

*Epi-pledget* Epinephrine compression, *Hb* hemoglobin, *AMH* anti-Mullerian hormone, *AFC* antral follicle count.

**Figure 1:**
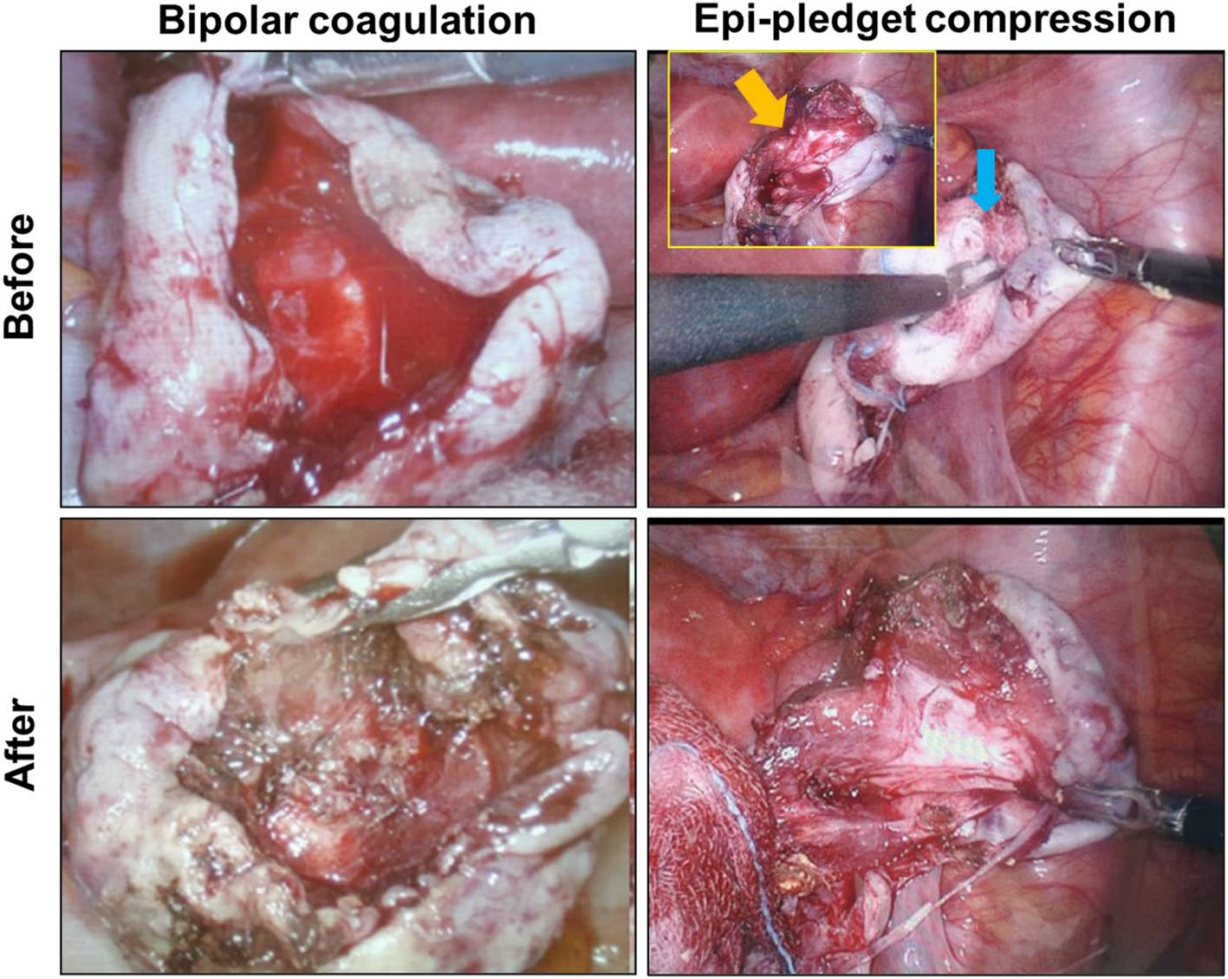
An operative finding of hemostasis on a laparoscopic ovarian cystectomy lesion. Hemostasis with epinephrine-soaked pledget compression (Epi-pledget compression) and bipolar coagulation before and after laparoscopic stripping of ovarian cysts. Yellow and blue arrows indicate bleeding of the laparoscopic cystectomy lesion and Epi-pledget compression on the stripping lesion, respectively.

Figure 1 illustrates tissue changes in representative cases after hemostasis using different methods. We observed thermal injury in the bipolar coagulation group (Fig. 1). The Epi-pledget compression successfully stopped bleeding without burn injury in the stripped lesion of laparoscopic ovariectomy (Fig. 1).

We measured the levels of AMH and AFC before and after surgery. Both the serum level of AMH and AFC count significantly decreased in the bipolar coagulation group compared with the Epi-pledget compression group and the Epi & Coagulation group (Table 2). Of note, no significant difference was observed regarding AMH and AFC between the Epi-pledget compression group and the Epi & Coagulation group (Table 2). We investigated the effect of hemostatic methods on AMH and AFC in patients stratified according to age. The group consisting of older subjects (age ≥ 34) showed a significant difference in AMH according to different hemostatic methods (Table 2). Among the subgroups of the four types of ovarian cyst, the differences of AMH and AFC decline were insignificant (Supplementary Table S2).

**Table 2:** Comparison of AMH and AFC decline rates between hemostatic groups.

	Types of hemostasis			P-value
	Bipolar Cauterization group	Epi-pledget group	Epi-pledget & Cauterization group	
**All subjects**	(n = 65)	(n = 62)	(n = 52)	
Rate of AMH decline (%)	24.5 (8.1–40.4)	0.8 (–18.7–39.2)^a^	10.34 (–17–30.0)^a^	0.005
Rate of AFC decline (%)	33.3 (– 41.7–60.0)^b^	25.0 (–100–50)	25.0 (–50–50)	0.026
**Age < 34 years**	(n = 42)	(n = 34)	(n = 31)	
Rate of AMH decline (%)	24.4 (7.5–40.6)	4.0 (–24.8–35.6)	20.5 (–11.1–43.3)	0.052
Rate of AFC decline (%)	33.3 (– 25.0–50.0)	29.2 (–50–45.8)	0 (–100–50)	0.125
**Age ≥ 34 years**	(n = 23)	(n = 28)	(n = 21)	
Rate of AMH decline (%)	27.1 (14.9–39.22)	– 2.4 (–8.5–40.5)	– 3.7 (–37.5–18.5)^a^	0.003
Rate of AFC decline (%)	50.0 (–41.7–63.3)	0 (–100–50)	33.3 (–50.0–50.0)	0.09

Data are presented as median (interquartile ranges).

Decline rate was defined as 100 x [preoperative level (AMH or AFC) – postoperative level (AMH or AFC)] / preoperative level (AMH or AFC).

*AMH* anti-Mullerian hormone, *AFC* antral follicle count, *Tukey HSD* Tukey honest significant differences.

^a^*p* < 0.05 vs. Bipolar coagulation group by Tukey HSD post hoc comparison.

^b^*p* < 0.05 vs. Epi-pledget compression group by Tukey HSD post hoc comparison.

Histopathological examination was conducted in all patient cases (n = 198), yielding the following diagnoses: benign ovarian cyst, either mature cystic teratoma (n = 75, 42%) or endometrioma (n = 69, 39%); or serous and mucinous cystadenoma (n = 35, 20%). No significant differences were evident in the mean maximum diameter of each ovarian cyst type [6.34 ± 1.7 cm (mean ± standard deviation) in the endometrioma group, 6.90 ± 2.0 in the teratoma group, and 6.72 ± 2.86 in the serous and mucinous cyst group]. Furthermore, the rates of decline in serum AMH and AFC did not differ among the subtypes of ovarian cyst (data not shown).

**Figure 2:**
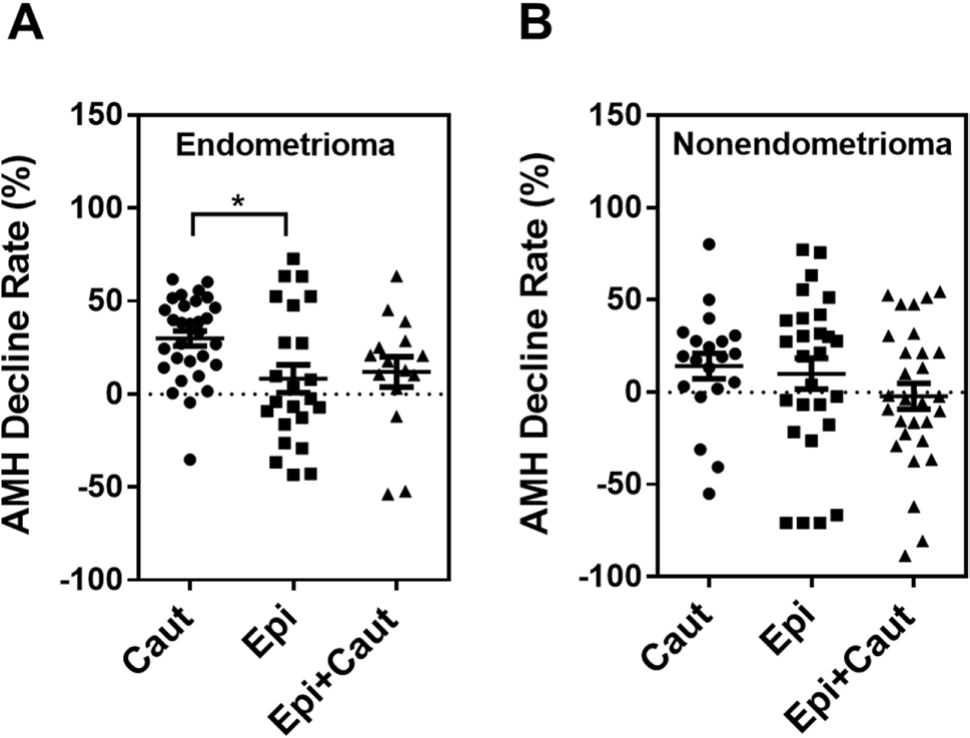
Comparison of AMH decline rates between hemostatic groups according to ovarian pathologic subtypes. AMH decline rates (%) in endometrioma (**A**) and non-endometrioma (**B**) groups. Decline rate was defined as 100 × [preoperative AMH level – postoperative AMH level] / preoperative AMH level. *p < 0.05. *Caut* cauterization, *Epi* epinephrine-pledget compression, *Epi + Caut* cauterization and epinephrine-pledget compression.

Next, we performed a subgroup analysis based on the endometrioma and non-endometrioma groups (Supplementary Table S1, Fig. 2). In the case of endometrioma, the Epi-pledget compression group showed less AMH decline than the coagulation group (Fig. 2A, Supplementary Table S1). When comparing the rate of serum AMH decline in the coagulation group, patients with endometrioma showed a greater AMH decline than those with non-endometrioma (Supplementary Table S2). The rate of AFC decline was not significantly different among the three hemostatic intervention groups and the ovarian pathologic cyst types.

**Figure 3:**
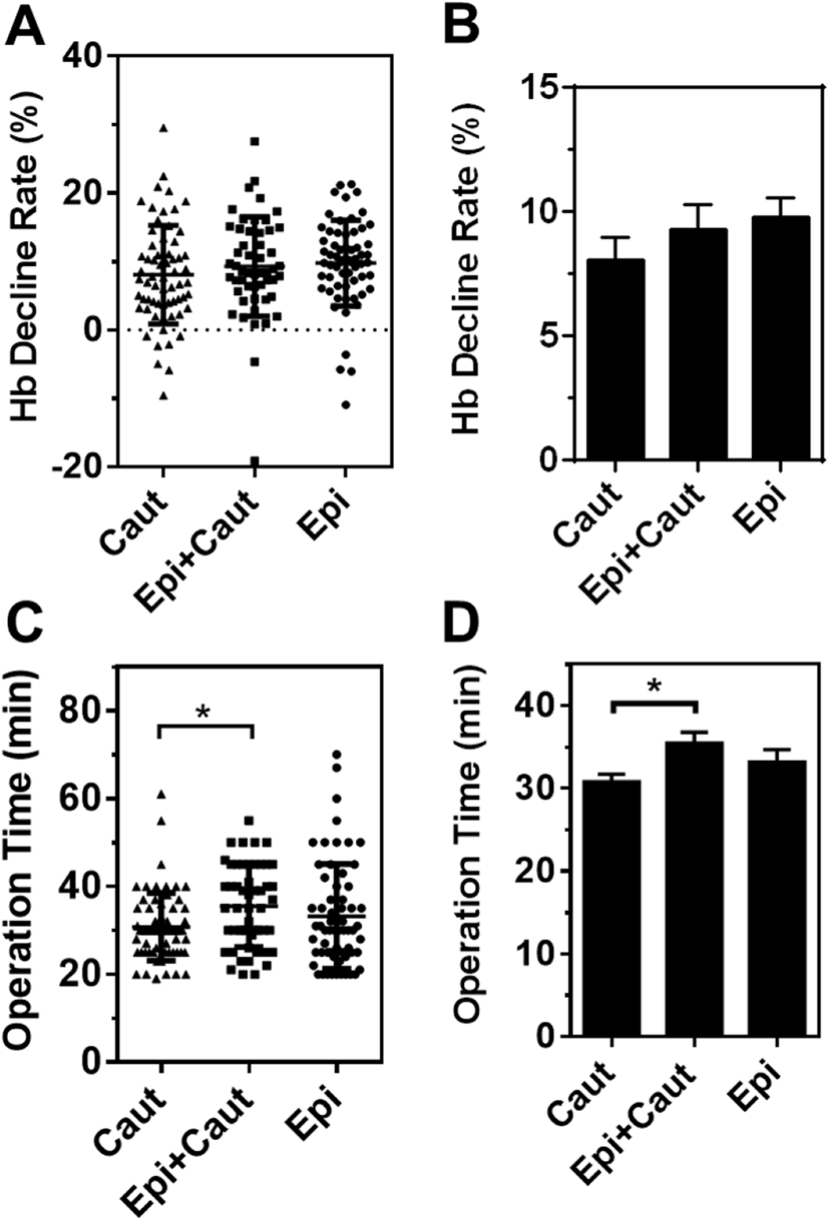
Postoperative change of hemoglobin and operation time. (**A**) Hemoglobin (Hb) decline rate in individual patients between hemostatic groups. Decline rate (%) was defined as 100 × [preoperative Hb level – postoperative Hb level] / preoperative Hb level. (**B**) Summary of panel A. (**C**) Operation time (min) of patients between hemostatic groups. (**D**) Summary of panel C. *p < 0.05. Data are presented as mean ± standard deviation. *Caut* cauterization, *Epi* epinephrine-pledget compression, *Epi + Caut* cauterization and epinephrine-pledget compression, *Hb* hemoglobin.

In hemoglobin changes and operative time based on the three hemostasis methods, we found that neither differed between the bipolar coagulation group and the Epi-pledget compression group (Fig. 3A–D). However, the coagulation group and the Epi & Coagulation group showed differences in operation time (Fig. 3C,D).

**Figure 4:**
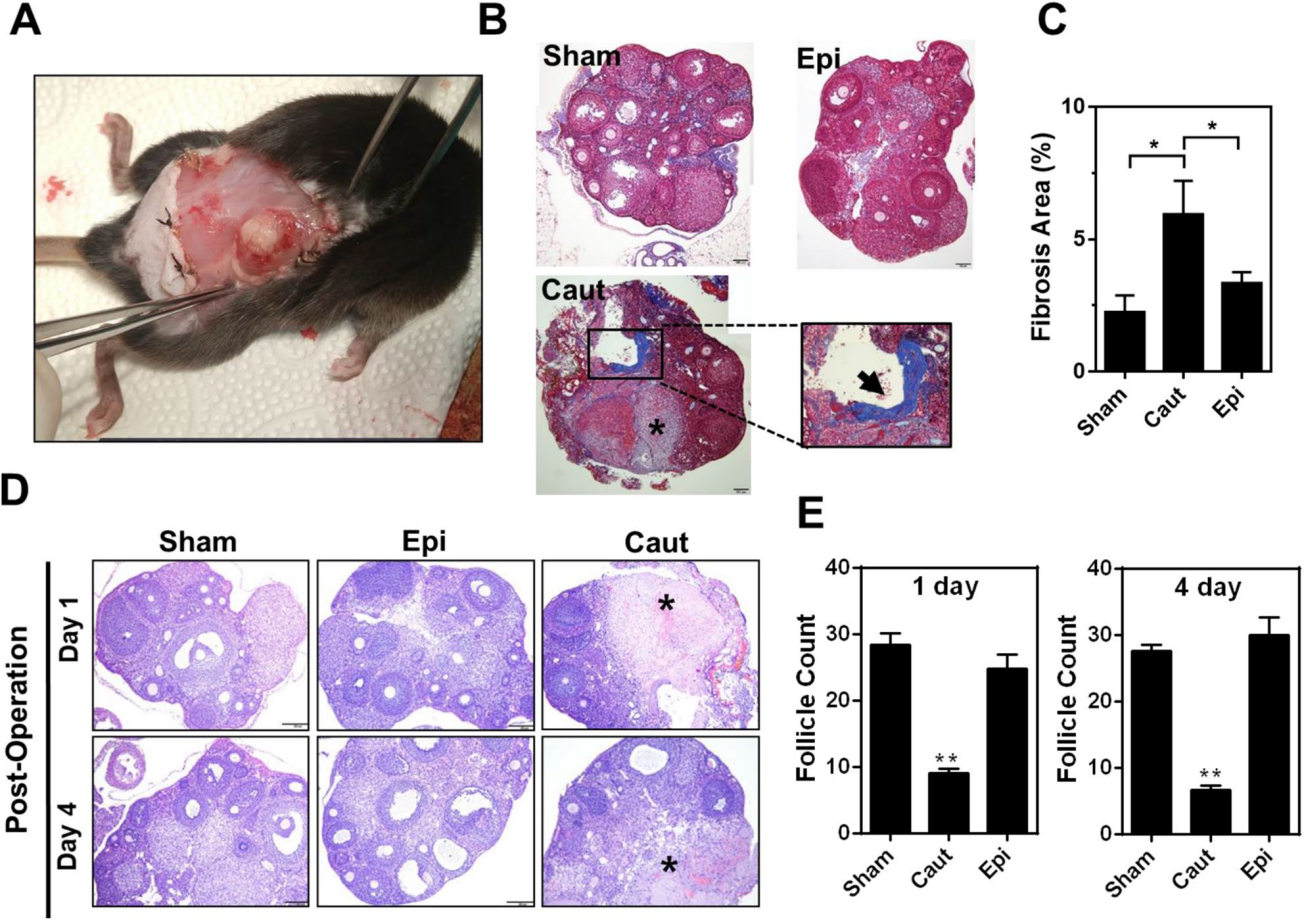
Quality of fibrosis and ovarian tissue reservation by hemostasis interventions in an injured ovary mice model. (**A**) Transverse incision at the lower back of the mouse and entry into the retroperitoneal space to approach the ovary. (**B**) Masson’s trichrome stain of mouse ovaries of each group (magnification, 200×). Necrosis region (*) and fibrotic tissue region (arrow, magnification, 400×) were found in the electrocauterization group. (**C**) Quantification of fibrotic area (%). *p < 0.05 (**D**) Histological evaluation of injured ovarian tissues after hemostasis with Epi-pledget compression and bipolar coagulation. The tissue of the Epi-pledget compression was almost similar to the sham group, whereas necrosis and fibrotic tissue change were observed in the coagulation group. An asterisk indicates necrotic regions. Magnification, 200×. (**E**) The follicles of each slide were counted to obtain the mean value. **p < 0.01. *Sham* normal ovary, *Caut* electrocauterization group, *Epi* epinephrine-soaked cotton pledget compression.

**Figure 5:**
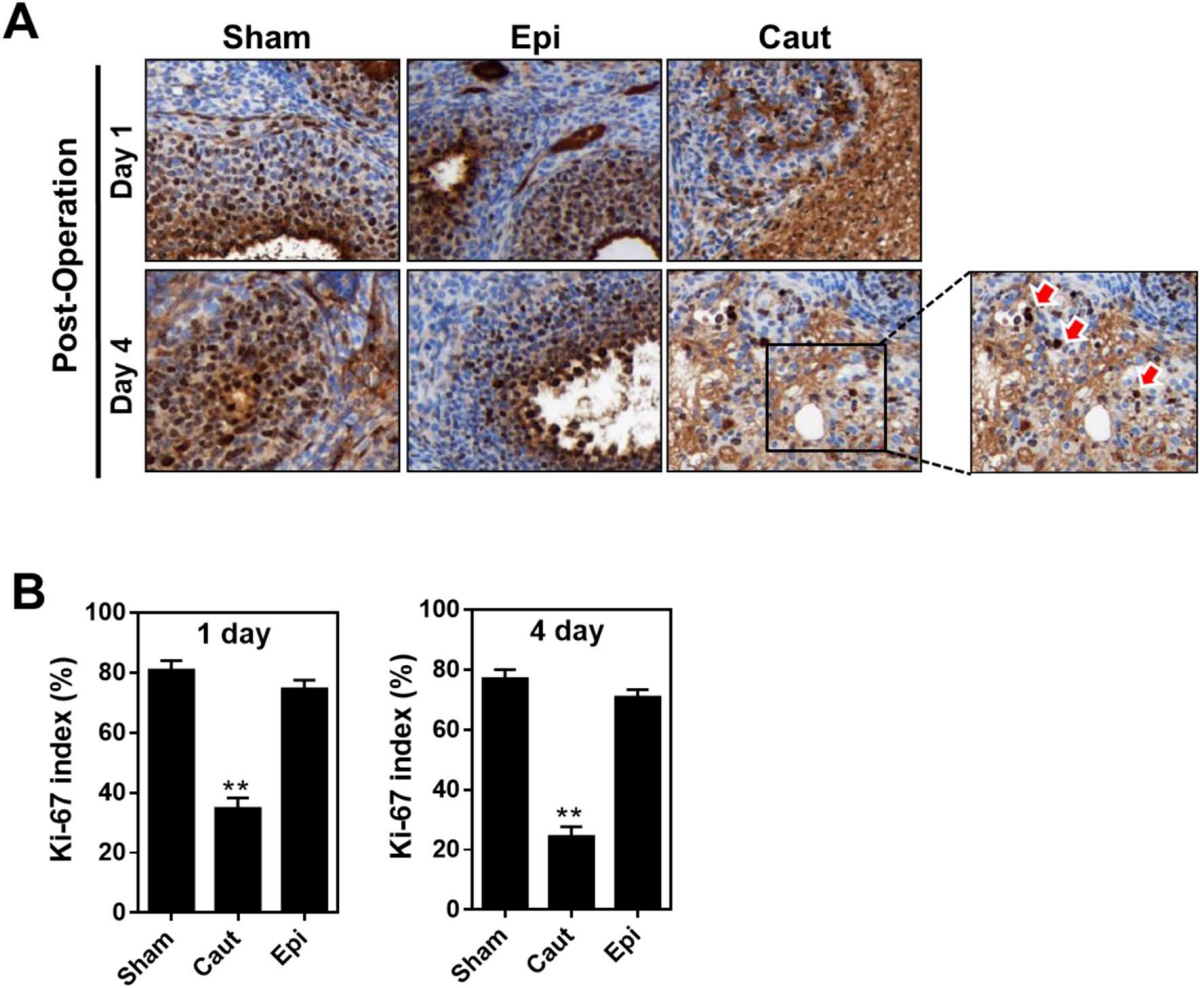
Proliferative capability by hemostasis interventions. (**A**) Proliferation capability was evaluated using Ki-67 immunoreactivity in mouse ovary tissue. Magnification, 200×. Stained nuclei (arrow) of the cauterization group ovarian tissue with Ki-67; the stained nuclei of normal growing follicular cells with Ki-67 (arrow) are easily identified. Magnification of inset, 400×. (**B**) Ki-67 index was calculated as the percentage of the number of stained nuclei at 1 and 4 days after the operation. **p < 0.01 vs. Sham group.

Together these results indicate that Epi-pledget compression is a favorable hemostasis intervention for the follicle reserve in patients. To examine pathological changes after applying hemostatic interventions, we employed a mouse model and evaluated acute and chronic changes in the injured ovary tissues after hemostatic interventions (Fig. 4A). No cardiovascular or other complications were observed in the mice, although the same concentrations of epinephrine (0.1%) were used in human patients. Electrocoagulation intervention exaggerated blood vessel collapse and fibrotic changes in mouse ovaries more so than epinephrine intervention (Fig. 4B,C). Moreover, electrocauterization caused a wide range of tissue injuries with eosinophilic granulomatous tissue formation around the necrotic area and a decrease in the number of growing follicles, while Epi-pledget intervention ameliorated the tissue and follicle injuries (Fig. 4D,E). Next, we examined the proliferative potential after hemostasis interventions using Ki-67, a proliferation marker. Ki-67 immunoreactivity in the granulosa cell layer of the growing follicle was markedly reduced in the electrocoagulation group, while the Epi-pledget intervention maintained Ki-67-positive cells (Fig. 5A). This was further confirmed by the Ki-67 index, which was significantly lower in the electrocoagulation group compared with the epinephrine group (Fig. 5B).

## Discussion

The present study demonstrates the beneficial effects of epinephrine pledget compression on an ovarian cystectomy lesion preserving ovarian reserve. Additionally, we also provide evidence that the Epi-pledget causes less thermal damage to the ovarian parenchyma than bipolar coagulation in an animal model. Electrocoagulation was shown to cause a greater decline in serum AMH and/or AFC after surgery than suture^19,20^. The hemostatic method in group 3 included both Epi-pledget and bipolar cauterization. In detail, the major hemostatic method in group 3 was the Epi-pledget and the bipolar cauterization was the assistant hemostatic method for oozing site after the application of the Epi-pledget. The Epi-pledget reduced the time of application of the bipolar cauterization and might alleviate the ovarian damage by electronic power. In hemostatic interventions according to the pathologic subtype of ovarian cyst, the rate of serum AMH decline was lower in the endometrioma group with bipolar coagulation than in the endometrioma group with Epi-pledget compression or Epi & coagulation. Animal experiments supported the notion that bipolar coagulation aggravated the reduction of ovarian reserve after surgery and caused irreversible ovarian tissue damage compared with that of the Epi-pledget. The primary pathophysiological mechanisms in irreversible ovarian tissue injury were inflammation and fibrosis that were induced by the thermal damage by electrocauterization rather than vasoconstriction and ischemic change by pharmacological intervention.

The suture had fewer adverse effects than bipolar coagulation in bleeding ovarian tissue after stripping^19,20^. Materials of gel or film type, such as elastin-thrombin matrix sealant, have also been proposed for hemostasis^21^. However, in patients with a past medical history of using hemostatic sealant during gynecologic surgery, adhesion to the adjacent adnexal organ or bowel obstruction has been reported^22^.

Vasoconstriction agents are widely used for hemostasis by the tourniquet effect on blood vessels^23^. Agents are injected directly into the bleeding lesion to constrict blood vessels, but these methods could cause a harmful reaction to the systemic circulation. Epi-pledget compression has been applied to the bleeding lesion in various other clinical settings^15^ and used as a reasonable choice to assist in securing the operative field. However, epinephrine itself could have cardiovascular effects^23^. The mechanism of cardiac muscle susceptibility to hypersensitivity is not well understood, but increases in arterial blood pressure, changes in heart rate, and increased cardiac muscle autonomy are factors affecting arrhythmia^24^. In this study, we conducted hemostasis by the 0.1% epinephrine-soaked pledget compression method, not an injection. Our results showed that during the operation of 114 patients with the Epi-pledget, no systemic cardiovascular problems occurred. Furthermore, there were no cardiovascular complications in animal experiments.

There were no significant differences in pre-postoperative hemoglobin changes and operation time between the bipolar coagulation and epinephrine intervention groups. Indeed, the advantages of electro-cauterization in laparoscopic ovarian stripping are a reduction in hemorrhage and a shorter operation time^23^. In the present study, we showed that simple Epi-pledget compression alone or assisted application of Epi-pledget compression could be comparable to the cauterization method. The Epi & Coagulation group had a significantly longer operation time than the coagulation group, but this did not affect the decline in AMH and hemoglobin levels. These results suggest that surgeons should consider the use of Epi-pledget compression alone or with minimal assistance of coagulation to preserve the ovarian reserve after laparoscopic ovarian cystectomy. Similar to our study, the Epi-pledget or epinephrine-involved intervention was applied in other fields of surgery or procedures^14–18^. The effectiveness of hemostasis and the preservation of normal tissue function of the Epi-pledget indicated the potential application at the surgery for other hormone-secreting tissues^25^.

The challenging issue in the surgical treatment of ovarian cysts is the possibility of encountering an unexpected ovarian malignancy. Approximately 0.9% and 3.0% of benign-appearing ovarian cysts were reported as unsuspected ovarian cysts in pre- and post-menopausal patients, respectively^26^. As another study recommended^26^, we strictly selected subjects via medical history, physical examination, serum CA125, and the transvaginal US. As a result, we did not report any patients diagnosed with ovarian malignancy.

There were several limitations to our study. First, the sample size of the endometrioma group was relatively small for showing the statistical significance of epinephrine effects on ovarian reserve levels. Further studies should increase the sample size and evaluate the quality of tissue healing to clarify the tissue changes according to various hemostatic methods, such as atraumatic suturing or various hemostatic sealants. Second, outcomes were measured over a relatively short 6–8-week follow-up period to determine the postoperative change in AMH levels or in tissue damages. Finally, our study compared the effects of coagulation with epinephrine. Further studies are needed to examine the hemostatic effects with ovarian reserve by various hemostatic sealants and vasocontraction agents such as vasopressin. Also, further comparison analyses between pharmacological interventions and suturing methods are required. Despite these limitations, our study provides unique strengths: a prospective design, inclusion of a variety of cyst types, and comparison of a new hemostatic method with established hemostatic methods concerning the preservation of ovarian reserve and pathologic subtype. Moreover, we used animal experiments to examine the pathologic changes in tissue after hemostatic surgery.

Our study reveals that hemostasis by Epi-pledget compression significantly increased ovarian reserve preservation compared with cauterization during laparoscopic benign ovarian cystectomy. Experiments using a mouse cystectomy model support the findings that the Epi-pledget intervention effectively ameliorates irreversible tissue damage causing the reduction of ovarian reserve. Based on the effectiveness and safety of bleeding control, the Epi-pledget showed similar effectiveness as bipolar coagulation. Our study suggests that Epi-pledget compression increases the preservation of the ovarian reserve by minimizing the use of coagulation.

## Methods

### Participants

According to the Consolidated Standards of Reporting Trials (CONSORT), we conducted a randomized controlled trial of patients who underwent laparoscopic ovarian cystectomy between January 2019 and September 2019 in the Department of Obstetrics and Gynecology at the Yonsei Wonju Severance Christian Hospital. The study was approved by the Institutional Review Board (IRB) of the Yonsei Wonju Severance Christian Hospital (CR118035). All procedures were conducted in adherence to the Declaration of Helsinki and CONSORT.

**Figure 6:**
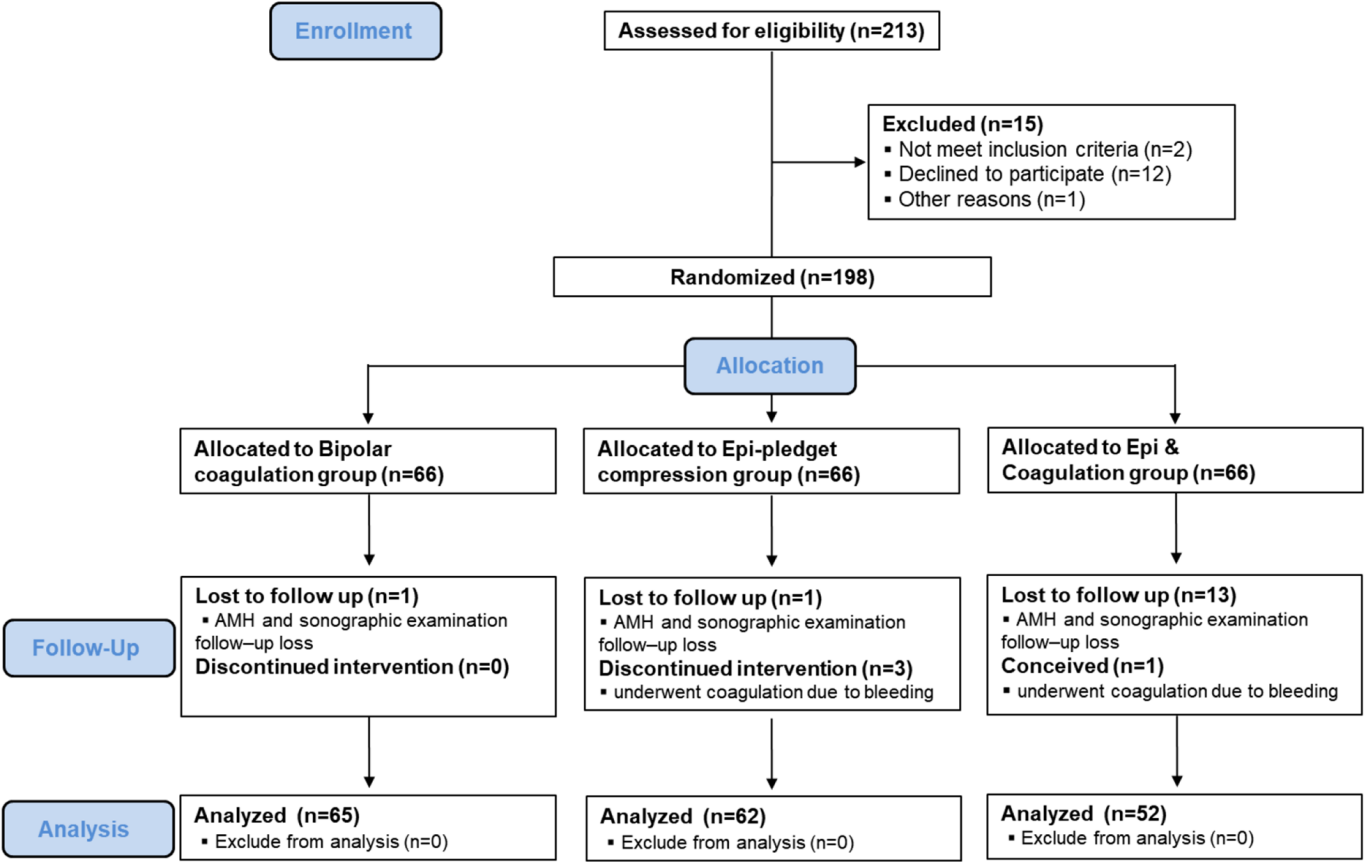
Study flow diagram showing the study protocol and sequence of events in this study.

Inclusion criteria were as follows: unilateral ovarian cyst diagnosed by ultrasound examination; diameter of the ovarian cyst between 3 and 15 cm; and appropriate medical status for laparoscopic surgery (American Society of Anesthesiologists Physical Status classification 1 or 2). In detail, we included operative subjects with symptomatic huge size of ovarian cysts. We operated on patients with pathologic ovarian cysts (endometriotic cyst, dermoid cyst, serous cyst, mucinous cyst) that had multi-septated and heterogeneous features diagnosed by transvaginal ultrasonogram (US). Exclusion criteria were as follows: evidence of any other endocrine disorder such as diabetes mellitus, thyroid dysfunction, hyperprolactinemia, and Cushing's syndrome; postmenopausal status; pregnancy; the use of any hormonal treatment (i.e., use of oral contraceptive pills in the three months before surgery); previous surgery due to adnexal pathology; any complication during operation; conversion to laparotomy; and loss of follow-up. We initially included 198 participants in this study, of which 179 participants completed the follow-up check. All patients provided written informed consent before participation (Fig. 6).

We named the epinephrine soaked pledget (hemostatic method) as Epi-pledget. We annotated the hemostatic method using the Epi-pledget and bipolar coagulation as Epi & Coagulation. Patients were randomly assigned to one of three groups: (1) bipolar coagulation, (2) Epi-pledget, and (3) epinephrine with coagulation (Epi & Coagulation), at a 1:1:1 ratio using a random permuted-block algorithm and a comprehensive internet-based randomization service for clinical trials available at http://www.randomize.net. The surgeon was not involved in group allocation. The patients were blinded to the hemostatic method to be used in their operation.

### Study protocol

The primary outcome of the present study was the reserve of ovary tissue and the function after laparoscopic ovarian cyst removal. The ovarian reserve was mainly checked by AFC and AMH. US examination was performed with the 5–6-MHz transvaginal transducer (SSD-α10, Aloka, Japan) to determine the unilateral ovarian cyst size and antral follicle count (AFC) of both ovaries. All subjects underwent transvaginal and/or transrectal US examination twice, once preoperatively and once postoperatively. The preoperative examination was done in the early follicular phase of the menstrual cycle (days 3–7). The postoperative examination was done on a similar day during the second menstrual cycle at 6–8 weeks. Using the US, the AFC was determined as the total number of follicles with a diameter smaller than 10 mm, and both ovaries were counted using the largest cross-sectional sagittal view of the ovary.

In all study groups, blood samples were taken within two weeks prior to and within 24 h after surgery. AMH levels were checked on the same day as US examinations. The decline rate of the serum AMH/AFC level (%) was calculated as follows: 100 × ([preoperative AMH/AFC level—postoperative AMH/AFC level] / preoperative AMH/AFC level).

### Operative procedure

Operative laparoscopy was performed in the usual manner described in several studies. After the entire cystic portion was separated from the ovarian cortex without vasopressin, a hemostatic material, bipolar coagulation, Epi-pledget compression, or Epi & Coagulation was applied for hemostasis. In the epinephrine groups, diluted epinephrine (0.5 mg/mL of epinephrine) in 50 mL of saline solution was used. In the Epi-pledget compression group, the bleeding sites were covered with the Epi-pledget and compressed directly with a laparoscopic applicator for 2 min to allow hemostasis (Fig. 1). In the Epi & Coagulation group, minimal electrical bipolar coagulation was performed after compression with the Epi-pledget for 1 min. All operations in this study were performed by one surgeon experienced in laparoscopic ovarian cystectomy (Dr. E. Y. Park). An electrocardiogram, used to detect arrhythmia, was performed before and after applying compression of the Epi-pledget. During compression of the Epi-pledget, systolic and diastolic blood pressure and heart rate were measured by an anesthesiologist. The subsequent procedures included careful inspection of the pelvic and peritoneal cavity by peritoneal washings, adhesiolysis for release, mobilization of the ovaries from the surrounding structures, and aspiration of the cyst material by a suction needle. A negative pressure drainage bag (Hemovac) was inserted into the cul-de-sac for enucleation of large-sized (> 10 cm) ovarian cysts and large volume irrigation (> 1000 cc). Hemostasis, on the ovarian cyst, stripped the oozing bed, was performed either by a 35–40 W current bipolar coagulation or through the application of an epinephrine-soaked cotton pledget. Total operative time was calculated from skin incision to skin closure. To confirm the diagnosis and exclusion of malignancy, the cyst wall was sent for histological assessment. None of the operated ovaries were sutured. The subjects were discharged from the hospital postoperative 2 or 3 days without postoperative fever and narcotic analgesics. All subjects were seen for follow-up at 6 to 8 weeks after surgery.

A complicated case was defined as when a patient experienced acute or delayed bleeding or epinephrine-related cardiopulmonary problems (dysrhythmias, hypotension, and pulmonary edema). We checked the degree of bleeding by comparing hemoglobin (Hb) levels from the blood test at the pre-operative assessment with those at 1 day and 6 to 8 weeks after surgery. There was no complicated case during the follow-up and we did not observe any anesthesiology problems. Furthermore, no patients experienced post-operative fever or infection of port site lesions.

### Animal model

Thirty female mice (C57B6/J) aged 12 weeks and around 21–23 g were randomly divided into three groups according to hemostasis techniques: the sham (no artificial incision injury and treatment to the ovary) group, the bipolar coagulation group, and the Epi-pledget compression group (n = 10 per group). After an operation was performed to create an artificial incision injury to the ovary, bipolar coagulation, and Epi-pledget compression techniques were applied to the injured ovary. Five mice in each group were sacrificed at postoperative day 1. The remaining five mice were sacrificed at postoperative day 4. Except for the sham group, the ovary wounds in all other groups were treated with hemostasis. The hair on the backs of the mice was shaved 2 days before surgery. Mice were anesthetized via inhalation followed by a transverse incision at the lower back of the mouse (Fig. 4A) and entry into the retroperitoneal space to approach the ovary. A partial incision of the ovaries resulted in wound production and bleeding of the ovary. To stop the bleeding, bipolar coagulation and diluted epinephrine (0.5 mg/mL of epinephrine in 50 mL of saline solution)-soaked cotton pledget compression were introduced. When the stoppage of bleeding was confirmed in the damaged lesion, the skin was closed by suture and the mice were checked for vital signs and complications at 1 and 4 days. All animal research protocols were approved by the Institutional Animal Care and Use Committee, Yonsei University Wonju College of Medicine, Korea (YWC-170622–1).

Histological evaluations of ovaries and determination of ovarian reserve. Ovaries were harvested and included normal and wounded areas. All tissues were fixed in 4% paraformaldehyde for 48 h at room temperature. After fixation, ovaries underwent dehydration in graded ethanol and xylene and then were embedded in paraffin. Sections were cut with a thickness of 5 μm and attached onto slides. The sections were deparaffinized in xylene and rehydrated in graded alcohol. After hydrating with deionized water, the sections were stained with hematoxylin and eosin (H&E), Masson’s trichrome (MTC) for detecting fibrosis, or Ki-67 for detecting proliferation. Stained sections were examined in a bright field with an Olympus microscope and photographed with a DP73 camera; the images were exported to CellSens Software. Fibrotic areas were measured and analyzed using Image J software (http://imagej.nih.gov/ij/imdex/html).

### Statistical analysis

Data were analyzed using Prism software (version 6, GraphPad Software, San Diego, CA, USA). Baseline clinical characteristics and study outcomes were compared among the three groups using a one-way analysis of variance (ANOVA) with multiple comparisons followed by Tukey’s multiple comparisons. *P-*values less than 0.05 were considered significant.

### Ethics approval and consent to participate

All procedures involving human participants were in accordance with the ethical standards of the institutional and/or national research committee and the 1964 Declaration of Helsinki and its later amendments or comparable ethical standards. Informed consent was obtained from all participants included in the study.

## Supplementary information

Supplementary Information
