# Supplementary material for: Epinephrine minimizes the use of bipolar coagulation and preserves ovarian reserve in laparoscopic ovarian cystectomy: a randomized controlled trial
Source: Sci Rep. 2020 Dec 1;10:20911. doi: 10.1038/s41598-020-77781-w (PMC7708492; doi:10.1038/s41598-020-77781-w)
Supplement: Supplementary file 1 — Supplementary Information [file 41598_2020_77781_MOESM1_ESM.docx]

(Supplementary File)

Epinephrine Minimizes the Use of Bipolar Coagulation and Preserves Ovarian Reserve in Laparoscopic Ovarian Cystectomy: A Randomized Controlled Trial

# Eun-Young Park^1^, Kyu-Hee Hwang^2^, Ji-Hee Kim^2^, San-Hui Lee^1^, Kyu-Sang Park^2^,

# Seong Jin Choi^1*^ & Seung-Kuy Cha^2*^

^1^Department of Obstetrics and Gynecology, Yonsei University Wonju College of Medicine, Wonju, Republic of Korea. ^2^Department of Physiology, Department of Global Medical Science, Mitohormesis Research Center, Institute of Mitochondrial Medicine, Yonsei University Wonju College of Medicine, Wonju, Republic of Korea.

Supplementary Table 1**.** Additional data for Table 1

|  | Bipolar Cauterization group (n=65) | Epi-pledget group  (n=62) | Epi-pledget & Cauterization group  (n= 52) | ***P value*** |
| --- | --- | --- | --- | --- |
| AMH level(ng/dL) |  |  |  |  |
| Preoperative | 5.16 (2.54-7.26) | 5.47 (2.6-7.66) | 3.90 (2.89-5.9) | 0.057 |
| Postoperative | 3.90 (1.90-6.13) | 3.91 (2.44-6.00) | 3.10 (2.15-4.93) | 0.083 |
| AFC(n) |  |  |  |  |
| Preoperative | 2.52 ± 1.08 | 2.21 ± 0.94 | 2.25 ± 1.04 | 0.211 |
| Postoperative | 1.66 ± 0.92 | 2.08 ± 0.91 | 1.94 ± 0.85 | 0.03 |
| Data are presented as mean ± standard deviation, median (interquartile ranges) or frequencies (percentages).  Epi-pledget, Epinephrine compression; Hb, hemoglobin; AMH, anti-Mullerian hormone; AFC, antral follicle count | | | | |

Supplementary Table 2. Comparison of AMH and AFC decline rates based on the type of ovarian cyst

|  | Types of ovarian cyst | | | | P-value |
| --- | --- | --- | --- | --- | --- |
|  | Endometriotic cyst | Mature cystic teratoma | Serous cystadenoma | Mucinous cystadenoma |  |
| All subjects | (n = 59) | (n = 88) | (n = 24) | (n = 8) |  |
| Rate of AMH decline (%) | 19.5 (–6.3 – 34.7) | 17.6 (–9.2 – 45.1) | 13.3 (–8.6 – 28.1) | 29.4 (–6.3 – 55.8) | 0.362 |
| Rate of AFC decline (%) | 33.3 (–75.0 – 50.0) | 33.3 (–50.0 – 50.0) | 50.0 (–62.5 – 50.0) | 25.0 (–62.5 – 62.5) | 0.762 |
| Data are presented as median (interquartile ranges).  Decline rate was defined as 100 x [preoperative level (AMH or AFC) – postoperative level (AMH or AFC)] / preopertive level (AMH or AFC).  AMH, anti-Mullerian hormone; AFC, antral follicle count | | | | | |

Supplementary Table 3. Comparison of AMH and AFC decline rates between hemostatic groups according to ovarian pathologic subtypes

|  |  | **Coagulation** | **Epi-pledget compression** | **Epi & Coagulation** |
| --- | --- | --- | --- | --- |
| AMH decline (%) | Endometrioma | 20.5%(15.3-48.1) ^a^ | 12.5%(11.4- 17.6*)* ^b^ | 17.7%(13.1-28.6) |
|  | Nonendometrioma | 16.5%(12.1-30.0) | 10.4%(9.1-19.7) | 15.3%(14.5-21.0) |
| AFC decline (%) | Endometrioma | 33.3%( -8.0-61.7) | 25.0%(-50-50.0) | 25%(-50-45.8) |
|  | Nonendometrioma | 33.3%(-50.3-50.7) | 33.3%(-87.5-50.0) | 33.3%(-75-50.0) |
| Data are presented as median (interquartile ranges) of frequency (%).  Decline rate was defined as 100 x [preoperative level (AMH or AFC) – postoperative level (AMH or AFC)] / preopertive level (AMH or AFC).  ^a^ p < 0.01 vs. Nonendomerioma in coagulation treatment, ^b^ p < 0.01 vs. coagulation in endometrioma.  Abbreviations: AMH, anti-Mullerian hormone; AFC, antral follicle count | | | | |
